# Health service providers' views on barriers and drivers to childhood vaccination of FDMN/Rohingya refugees: a qualitative study in Cox's Bazar, Bangladesh

**DOI:** 10.3389/fpubh.2024.1359082

**Published:** 2024-07-09

**Authors:** Sarah Reda, Heide Weishaar, Sadika Akhter, Basel Karo, Jorge Martínez, Aarti Singh, Cath Jackson

**Affiliations:** ^1^Centre for International Health Protection, Robert Koch Institute, Berlin, Germany; ^2^School of Health and Social Development, Deakin University, Burwood, VIC, Australia; ^3^International Centre for Diarrhoeal Disease Research, Bangladesh (ICDDR,B), Dhaka, Bangladesh; ^4^World Health Organization Emergency Sub-Office, Cox's Bazar, Bangladesh; ^5^Valid Research Ltd, Wetherby, Leeds, United Kingdom

**Keywords:** vaccination, childhood, refugee, health worker, Rohingya, forcibly displaced Myanmar nationals (FDMN), Cox's Bazar, qualitative study

## Abstract

**Background:**

Despite established vaccination programs, vaccine-preventable diseases persist among about 900,000 Forcibly Displaced Myanmar Nationals (FDMN)/Rohingya refugees in the world's largest refugee settlement in Bangladesh. Health service providers (HSPs) play a key role in the delivery of childhood vaccination programs. This study explored their views on individual and context barriers and drivers to childhood vaccination in this setting.

**Methods:**

Informed by the theoretical framework of the Capability-Opportunity-Motivation-Behavior (COM-B) model for behavior change, this qualitative study collected data through eight focus group discussions (FGDs) with community health workers (CHWs) and vaccinators in selected camps with high or low vaccination coverage rates, and through 11 in-depth interviews (IDIs) with key informants working in strategic, management, and administrative roles.

**Findings:**

Barriers and drivers were evident across all COM factors for HSPs and caregivers. Among HSPs, knowledge around vaccination acted both as a barrier and driver, while communication skills and confidence in vaccination served as drivers. Caregivers' lack of awareness of vaccination, concerns and mistrust were described as main barriers. Context barriers included information system deficiencies, family dynamics, HSPs' working conditions, and vaccination site accessibility. Context drivers included effective communication, mobilization, and incentives. Differences between high and low coverage camps in Cox's Bazar included variations in HSPs' knowledge, communication strategies, incentive use, and stakeholder collaboration.

**Discussion:**

For better vaccination coverage in the camps, context-related changes regarding collaboration, health workforce and the use of incentives seem necessary. Caregivers' mistrust toward vaccination needs to be considered under the social and historical background of the Rohingya community, and further addressed with targeted communication and campaigning.

## 1 Introduction

Vaccination is one of the fastest, most cost-effective and lifesaving public health measures to date, showing its significance especially in the context of refugee populations who often live in challenging environments with higher risks of disease outbreaks (1, 2). Since 2017, almost one million Forcibly Displaced Myanmar nationals (FDMN)/Rohingya refugees have sought temporary shelter in the densely populated refugee camps of Cox's Bazar, Bangladesh (see Box 1). Despite an established routine immunization program for children up to 2 years of age, the community faces recurring vaccine-preventable disease (VPD) outbreaks (2), highlighting the complexities of immunization delivery in this setting.

Box 1 **Rohingya Refugee Crisis in Bangladesh.**

The Rohingya are a Muslim minority from Myanmar. During past decades, repeating waves of ethnic violence forced them to seek refuge in neighboring countries (3). The Rohingya crisis commenced in 1982 when Myanmar denied them citizenship, rendering them stateless and thus subject to violence, persecution, and rights denial, including restrictions to healthcare and education (3). Violence came to a peak in 2017, when ~700,000 Rohingyas fled to seek refuge in Bangladesh at once (4). Today, nearly one million (965,467) Rohingya refugees/Forcibly Displaced Myanmar Nationals (FDMN) are residing in the world's biggest refugee camp in Cox's Bazar areas of Ukhia and Teknaf, as well as on Bhasan Char Island (5). 52% of them are children (5).

Over the past years, the vaccination landscape in Cox's Bazar has undergone significant changes (6): As the Rohingya community was previously excluded from essential health services in Myanmar, about 60% of their children were unvaccinated when arriving in Bangladesh (7). After a first large-scale diphtheria outbreak shortly after the influx of Rohingya refugees/FDMN in 2017 (8), comprehensive vaccination campaigns were implemented, e.g., for measles (9). Internal data of the WHO shows that over the past years, coverage rates for fully vaccinated children have improved substantially from 37% in 2020 to 60% in 2022. Still, rates for certain VPDs like measles [with 60% coverage for the first vaccine dose (9)] never achieved to protect from emerging outbreaks.

An extensive body of global evidence shows that the underlying causes of under-vaccination are multifaceted, operating both at context and individual levels (10). The impact of a challenging context is arguably greater for refugees living in camps where infrastructure and resources are lacking (11–15). Specific to Cox's Bazar, a recent scoping review of 18 articles exploring barriers and drivers to FDMN/Rohingya refugees receiving childhood vaccination by Yusuf et al. (16) highlighted the importance of easy access to vaccination sites and significant influence of health service providers (HSPs), as well as community and gender-related norms on vaccination behaviors. At the individual level, caregivers' lack of knowledge about the purpose and availability of vaccination, alongside safety concerns about vaccines, religious beliefs and lack of trust acted as barriers to children receiving vaccination.

A key finding of the review was the lack of research in understanding vaccination from the HSPs' perspective (supply side). Indeed, of 18 studies included in the review, only two focused exclusively on HSPs and vaccination. Other studies (n=8) included HSPs alongside FDMN/Rohingya refugees, yet typically had a broader healthcare focus and/or did not use qualitative methods for detailed insights. In short, most research so far has focused on the perspectives of FDMN/Rohingya refugees (demand side). However, to fully understand why a vaccination programme does or does not function well, it is necessary to consider both demand and supply side factors (17). Beyond the administrative side of delivering vaccination, the importance of HSPs' recommendations for caregivers' vaccination decisions is well documented (18, 19): HSPs play a key role in trust building, and this is particularly important for refugee communities who have often fled persecution and violence in their home country (20, 21), may distrust their host country's health systems and typically face language barriers (11).

In light of this information gap, the study presented in this paper explored individual and context barriers and drivers to delivering childhood vaccination to FDMN/Rohingya refugees living in Cox's Bazar from the perspective of HSPs. It was part of a larger mixed-methods project that also included a survey with FDMN/ Rohingya refugee caregivers. By exploring different perspectives on childhood vaccination in Cox's Bazar, we aim to inform targeted and tailored interventions to enhance vaccination coverage for FDMN/Rohingya refugees, contributing to improved health in this setting prone to disease outbreaks.

## 2 Methods

We conducted a cross-sectional qualitative study focused on exploring the experiences and perspectives of HSPs on barriers and drivers to delivering routine childhood vaccinations of FDMN/Rohingya in Cox's Bazar. The study was conducted with frontline HSPs (community health workers (CHWs) and vaccinators) and key informants with strategic, management, administrative or partnership roles in childhood vaccination. The focus of the study, delivering vaccination, was broadly defined, and included conversations with caregivers, mobilizing communities, scheduling appointments, administering vaccinations as well as ensuring vaccine supply and cold chain. Details on how vaccination is organized in Cox's Bazar are given in Box 2.

Box 2 **How vaccination is organized in the refugee camps of Cox's Bazar.**

The makeshift settlements in Cox's Bazar are structured into 33 single camps for better camp management, including health service provision. The camps vary in size and accessibility, are clearly separated and comparable to self-organized districts with their own blocks and sub-blocks. Especially during the COVID-19 pandemic, movement between individual camps was extremely limited. The basic vaccination services of each camp are similar, including fixed vaccination sites (health posts, primary healthcare centers and specialized field hospitals) and community outreach posts.

Different professional groups are involved in vaccination activities. Community health workers (CHWs) from the refugee and local host population are actively involved in counseling community members and supporting service provision. Vaccinators are specially trained and mostly local people from the host population. Each health facility has a manager, overseeing health service provision. Additionally, the community-based Camp-in-Charge (CiC) group in each camp and ~15 immunization partners who work across the camps (including BRAC, UNHCR, UNICEF and IOM) support vaccination activities, amongst other things.

Childhood vaccination programs in Cox's Bazar refugee camps follow the schedule of the Expanded Program on Immunization (EPI) (22).

(Source: Personal communication with representative of WHO Sub Office, Cox's Bazar)

The study received ethical approval from the Institutional Review Board (IRB) of the Cox's Bazar Ethics committee, the Research Review Committee Bangladesh at WHO Bangladesh as well as WHO South-East Asia (SEARO).

### 2.1 Theoretical framework

**Figure 1:**
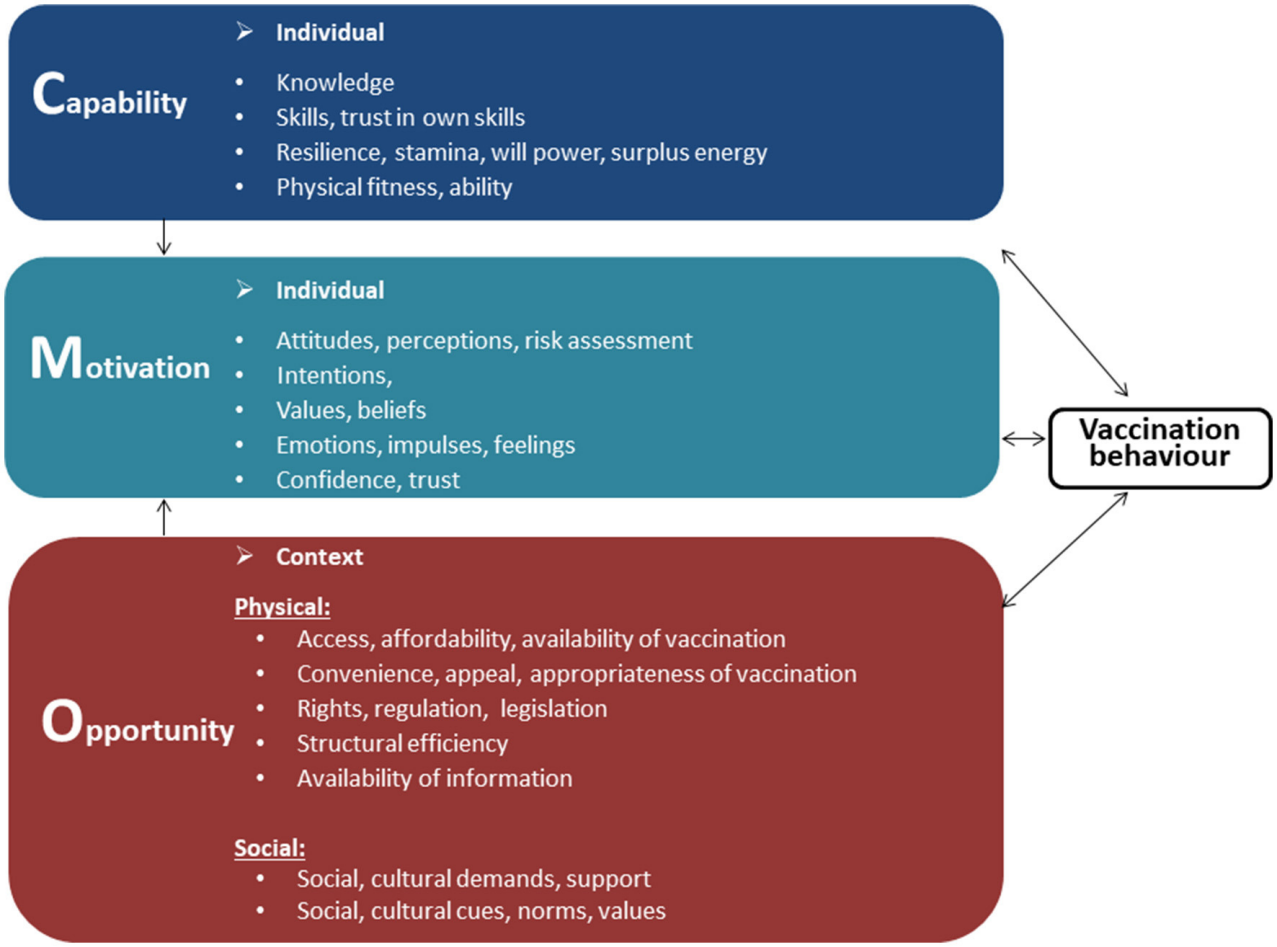
Modified COM-B model for vaccination behaviors (24).

The theoretical framework underpinning the study was the modified COM-B model (see Figure 1) (23, 24) which identifies the inter-linked factors of capability (knowledge, skills), physical opportunity (information, access, health systems), social opportunity (support, norms) and motivation (attitudes, confidence, trust) as influencing vaccination behaviors. This model informs the WHO Tailoring Immunization Programmes approach (17, 24) and has previously been used to understand the barriers and drivers to health workers delivering vaccination (25–27). It ensures that all potential individual and context barriers and drivers are considered, leaving no “blind spots” (24). Using the model also facilitates the process of linking barriers and drivers to evidence-informed interventions (17).

### 2.2 Study setting

The study was located in the two main settlements of Cox's Bazar: Ukhia (which has 26 camps) and Teknaf (which has seven camps). The camps were purposively sampled to ensure a mix of camps with high and low childhood vaccination coverage. The selection was done based on WHO concurrent monitoring data, using the third pentavalent vaccination (Penta 3) as an indicator with 22%−34% coverage for the low- and 93%−96% coverage for the high coverage camps.

### 2.3 Participants and recruitment

We recruited frontline HSPs (20 vaccinators and 40 CHWs) to participate in eight focus group discussions (FGDs). We used purposive sampling to ensure they were from different health facilities (health posts, primary healthcare centers, specialized field hospitals) and community outreach posts. A WHO staff member (AS) visited the selected health facilities/outreach posts, informed the manager about the study and requested them to nominate a vaccinator and a CHW to be invited to participate. Those who agreed to take part were booked into one of the FGDs. Three CHWs and two vaccinators declined to participant for reasons of sickness, maternity leave, and competing work priorities. They were replaced with willing HSPs.

A total of 11 in-depth interviews (IDIs) were conducted with key informants working on health service provision at camp or central level in Cox's Bazar in strategic, management, administrative or partnership roles. These included district and sub-district level government health officials (*n* = 5), government officials from the Ministry of Health and Family Welfare (*n* = 2), and NGO representatives (n = 5). AS contacted these key informants to discuss the study and share the study information. Those who agreed to participate were scheduled for an IDI. No one declined.

### 2.4 Data collection

Semi-structured topic guides informed by the COM-B model were used for FGDs and IDIs (see Supplementary material). These explored participants' knowledge of vaccination coverage/disease outbreaks, views on benefits and risks of childhood vaccination, vaccination procedures (adapted for different participant groups e.g., promoting, discussing vaccination with caregivers, mobilizing families, cold chain management), reasons for under-vaccinated children, and ideas for improving coverage. Topic guides were slightly amended based on the roles of the participants in the FGDs/IDIs. The FGD topic guides were piloted with vaccinators and CHWs in one camp, after which some re-ordering and small changes to the wording of some questions were done to improve flow and clarity. The IDI guide was not piloted, instead it was reviewed for improvement in clarity and flow after the first interview.

FGDs and IDIs were conducted in the local dialect by a Bangla-speaking, highly experienced, public health qualitative researcher (SA, PhD) accompanied by a WHO staff member (AS). Neither knew the participants. They were trained and supervised throughout by a senior researcher (CJ). Vaccinators were able to move around freely across camps at the timepoint of data collection, so they participated in cross-camp FGDs in the local WHO office. CHWs as part of the FDMN/Rohingya refugees were restricted to stay in one camp meaning that FGDs with CHWs occurred in a health facility inside the camps. IDIs were conducted online or face-to-face in the key informant's workplace. All FGDs and IDIs were audio-recorded and field notes were made at the end. Data collection was completed in December 2022 and February 2023. Each FGD and IDI lasted on average 1 h and 30 min respectively.

Before the start of the FGD/IDI, the purpose, funder, and organizations conducting the research were explained to participants. Written informed consent to participate and be audio-recorded was then collected Participants were informed of their right to withdraw from the study at any time without repercussions. In order to maintain confidentiality, all data went through a de-identification process, which involved anonymizing the field notes, transcripts, and audio recordings. Generic terms like “study participants” were employed by the researcher in place of using participants' names or very specific roles.

### 2.5 Data analysis

The data were analyzed using a rapid approach to qualitative data analysis (28, 29) to enable the findings to be promptly used to design tailored interventions. This method draws upon Framework analysis (30) which is designed to address policy and programme-related questions. Instead of producing verbatim transcripts, data from the audio recordings are directly organized into Microsoft Excel Rapid Assessment Procedure (RAP) sheets for analysis. The qualitative analysis team were the data collector (SA), WHO staff member (AS), and RKI scientists (SR, HW and colleagues). CJ provided training and supervision as senior researcher.

There were five steps for analysis. At every step, checks for inter-researcher consistency were done. First, CJ developed RAP sheets from the topic guides for each participant group (see Supplementary material for an example RAP sheet), structured by the COM-B model and to facilitate comparisons of high vs. low coverage camps. These were checked by SR. Next, SA listened to the audio-files in Bangla, paraphrased the data and inserted verbatim quotes into the RAP sheets in English. These were checked in Bangla by AS including checks for technical correctness. The RKI team then worked in two pairs to examine the data within each RAP sheet, and to compare and contrast views within each participant group, i.e., across high and low coverage camps. These researchers worked independently then came together in their pairs to jointly complete tables with descriptive findings for each participant group. A sub-sample (25%) of the tables was checked against the RAP sheets by CJ. The final step was to write up the barriers and drivers to delivering childhood vaccination, organized by the COM factors, triangulating the findings across the participant groups. The qualitative analysis team met to review and agree the final findings.

## 3 Findings

### 3.1 Participants

Table 1 provides an overview of participants in FGDs and interviews and respective camps, when applicable. Five vaccinators from 13 camps and 10 CHWs from four camps in Ukhia and Teknaf participated in four FGDs each. High and low coverage camps (HCC and LCC) were almost equally represented. Interviews were conducted with 11 government- and NGO representatives without focusing on particular camps.

**Table 1:** Overview of participants.

	**Focus group discussions with frontline HSPs**		**In-depth interviews with key informants**	
**Participants**	**Vaccinators**	**Community health workers**	**Government representatives**	**NGO representatives**
Number of participants	20	40	6	5
Number of focus groups/interviews	4	4	6	5
Number of camps covered	13	4	n.a.	n.a.
Types of camps covered	Ukhia: 3 HCC, 4 LCC, Teknaf: 3 HCC, 3 LCC	Ukhia: 1 HCC, 1 LCC, Teknaf: 1 HCC, 1 LCC	n.a.	n.a.

### 3.2 Views of HSPs

HSPs' views on the barriers and drivers to delivering vaccination are summarized in Table 2 and described here, organized by the four COM factors. For each factor, views on their own role (supply side) in delivering vaccinations are presented first, followed, where available, by their perceptions of caregivers' roles (demand side) in effective delivery. Very few differences were found between participant groups or high/low coverage camps so we usually refer to participants overall. Where there were differences, these are described. Illustrative quotes are presented throughout.

**Table 2:** Barriers and drivers to delivering childhood vaccination organized by the COM factors.

**COM factor**	**Barriers and drivers**
Capability	• HSPs' knowledge of childhood vaccination coverage, schedules and VPDs 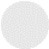 • HSPs' knowledge of purpose of vaccination 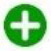 • Communication skills of frontline HSP 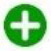 • Caregivers' awareness and understanding of vaccination 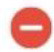
Physical opportunity	• Lack of fully-functioning information system on vaccination coverage and VPDs 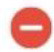 • Human resources and working conditions of frontline HSPs 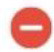 • Vaccine supply and cold chain 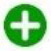 • Vaccination cards 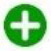 • Mobilizing and incentivising caregivers 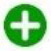 • Communicating with caregivers about vaccination 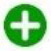 • Access to, and treatment at, vaccination sites 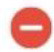
Social opportunity	• Communication and coordination among frontline HSPs and other stakeholders involved in vaccination 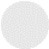 • Collaboration with community leaders 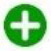 • Relationship between frontline HSPs and the community 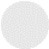 • Family dynamics
Motivation	• Positive attitude of HSPs toward vaccination 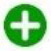 • Caregivers' concerns and lack of trust 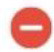

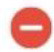
, barrier, 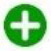, driver, 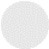 neutral (can act both as barrier and driver). Categorization as a barrier, driver or both (neutral) was based on the majority view amongst participants.

#### 3.2.1 Capability

The capability barriers and drivers related to HSPs' knowledge, their communication skills and perceptions of caregivers' understanding of vaccination.

##### 3.2.1.1 Knowledge of childhood vaccination coverage, schedules, and vaccine-preventable diseases

Vaccinators and government informants demonstrated varying levels of knowledge regarding childhood vaccination coverage across professional groups (there was no data on knowledge of coverage from CHWs). They stated a wide range of vaccination rates and estimates of the absolute number of fully vaccinated children by 2 years, with some (incorrectly) reporting that almost all children were vaccinated. Vaccinators from high coverage camps were overall better informed of their camp coverage rates than those from low coverage camps. This appeared to be due to them having structured processes in place for checking coverage and dropout rates. NGO representatives' estimates of 40%−50% coverage for Cox's Bazaar were closest to official statistics. Knowledge of the childhood vaccination schedules for Cox's Bazar and Bangladesh also differed across professional groups. Frontline HSPs showed to be aware of occurrence of vaccine-preventable diseases (VPDs) as they identify them during household visits and report to health authorities.

##### 3.2.1.2 Knowledge of purpose of vaccination

Overall, participants demonstrated good knowledge of the purpose of vaccination, describing its role in preventing various diseases, and commenting on the safety of vaccines. Notably CHWs and vaccinators from high coverage camps provided more specific examples of VPDs and the risks of not vaccinating, such as the potential for death or disabilities, compared to those working in low coverage camps. Only government and NGO representatives additionally mentioned that vaccination of FDMN/Rohingya refugees contributed to protecting the health of the host (Bangladeshi) community.

Participants described using a variety of different vaccination information sources. On-the-job training was mentioned as a key source for CHWs and vaccinators. Also, WHO publications and the media (e.g., TV, radio, social media) were cited as important sources for CHWs, vaccinators and NGO informants. Scientific publications were mentioned only by government and NGO representatives.

##### 3.2.1.3 Communication skills of frontline HSPs

Participants were unanimous about the importance of communication with caregivers which, they believed when done well, greatly facilitates vaccination uptake. CHWs and vaccinators reported that, overall, they feel confident and competent in these vaccination conversations. They described how they explain the benefits of vaccination and the risks of not vaccinating, informing caregivers of potential side-effects, using positive examples of how vaccination protects against diseases as well as fear appeal:

“*If you do not vaccinate your children, they might die. You will need to run from hospital to hospital.”* (CHW, Teknaf, high coverage camp)

They also reported conducting regular information sessions on vaccination for caregivers and family members, emphasizing how they almost exclusively communicate orally with caregivers in the local language to be more personable and address any hesitation. CHWs from high coverage camps further described how they check mothers' understanding by asking them to repeat the information back to them. It was suggested by government informants to develop standards for vaccination communication which could further improve interactions between frontline HSPs and caregivers.

“*I always describe the benefits of vaccines to caregivers. I explain that by administering vaccines to their children, they can protect them from ten dangerous diseases. I emphasize that vaccines help develop the body's ability to prevent diseases. I communicate with mothers in their language to ensure they understand the benefits of vaccination. Additionally, I reassure them that while their child may experience temporary side effects like fever, pain, and swelling, there are no serious adverse effects associated with vaccines, and it's normal for such reactions to occur.”* (CHW, Ukhia, high coverage camp)

##### 3.2.1.4 Caregivers' awareness and understanding of vaccination

There was a clear opinion amongst participants that some caregivers lack awareness of the importance and benefits of childhood vaccination. Furthermore, false information and associated misperceptions about vaccination were seen to hamper caregivers' willingness to get their children vaccinated. Examples of these were death and infertility following vaccination, or that the vaccination itself makes the children sick.

“*You (CHW) said that you are giving vaccines to our children to keep them healthy but our children are getting sick after taking vaccine.”* (CHW citing a caregiver, Teknaf, low coverage camp)

CHWs reported that there was a drop-out when there are long intervals in the vaccination schedule, specifically after the last pentavalent vaccination (Penta 3) until the first, and then the second dose of measles-rubella (MR) vaccination.

A positive finding was the general view that misconceptions related to religious beliefs had been significantly minimized via major campaigns run in Cox's Bazar. These beliefs, e.g., that the vaccine would convert them to another religion, had previously constituted significant barriers to vaccination.

“*In the past, they believed there would be a mark in the arm after taking the vaccine, which is a sign of Christianity. This belief does not exist anymore.”* (CHW, Teknaf, low coverage camp)

#### 3.2.2 Physical opportunity

Physical opportunity barriers and drivers related to information systems, human resources and working conditions, vaccine supply and cold chain, vaccination cards and incentives, mobilizing caregivers, opportunities for communication about vaccination, and access to vaccination sites.

##### 3.2.2.1 Information systems on vaccination coverage and vaccine-preventable diseases

In the absence of a fully functioning electronic information system, participants described relying on paper records (tally sheets) to track the steps in vaccination delivery such as the number of vaccines delivered from health facilities to vaccinators, and the number of vaccines administered to children or returned to the health facilities. Frontline HSPs were using paper-based records of children's vaccinations and sharing written lists of eligible children for vaccination and of children who had dropped out from vaccination. There was a general view that these data (and corresponding coverage data) are unreliable due to the lack of careful record-keeping and monitoring, including inaccurate lists of eligible children and duplication of records. Indeed, it was suggested by NGO partners that some of the variation in vaccination coverage between camps may be due to data problems i.e. not real differences.

“*There is a problem with the denominator. There is no correct information about the exact number of children. We do not have accurate data about the coverage. The data relating to the vaccine are collected from UNHCR, but their data is not up to date. I do not know the exact number of the fully vaccinated children. We do not have accurate line listing data. We have aggregate-level data.”* (NGO representative)

Participants further lamented that no robust VPD surveillance system exists and thus little to no official data on VPDs are available. Instead, information on VPD cases is shared informally between them. Specifically, CHWs, vaccinators and doctors who observe VPDs and VPD-related symptoms during household visits or clinics report this to the WHO health monitoring officer who formally records the VPD cases. NGO informants said they are then notified through field staff, clinics, monthly meetings, the WHO team and the emergency warning and reporting system.

##### 3.2.2.2 Human resources and working conditions of frontline HSPs

There was widespread acknowledgment that many CHWs and vaccinators have high workloads and are overburdened. Whilst workload varies across the camps, some CHWs may visit 100–200 households per week and be required to counsel families on a variety of health issues, not just vaccination. Similarly, vaccinators may have up to 4–5 blocks to visit in 1 day.

“*Camp 19 is so big, and it is not possible for one vaccinator to visit all households of 3 to 4 or 4 to 5 blocks for counseling in 1 day. We have said many times to increase the number of vaccinators for our camp.”* (Vaccinator, Ukhia, low coverage camp)

Key informants mentioned a lack of supervisory staff which they believed reduces the quality of the frontline HSPs' work and is a reason for variation in vaccination coverage across camps.

“*There is no supervision of the vaccinators from the government. The supervisors are overburdened to supervise the immunization work of the host community. They are assigned to do additional work to supervise the FDMN community work. A total of 132 vaccinators are working in the FDMN community, and there are 5–6 supervisors. How can only 5–6 supervisors supervise the 132 vaccinators?”* (NGO representative)

They also spoke specifically about the difficult working conditions of vaccinators. These included having nowhere to store materials e.g., vaccine supplies and the carriers they use to transport the vaccines, no designated vaccination area or chairs in the health facilities, and only receiving a salary every 4–5 months with no recent pay increases.

“*I walk with vaccine carriers, covering distances of 3 to 4 miles from the vaccine delivery point to the vaccine outreach center. Carrying heavy loads adds to the challenge. I do not have chairs to sit in and work long hours to provide vaccines. I also visit door to door to counsel the mothers without having rest. Our salary is not increasing, and I do not receive any benefits except salary. I sometimes get demotivated from working in these working conditions.”* (Vaccinator, Teknaf, high coverage camp)

Ideas to address the workload burden focused on engaging CHWs who solely focus on immunization, eliciting support from volunteers and community mobilisers, and recruiting vaccinators from the local FDMN community known to local families to have opportunistic conversations about vaccination, for example at the tea stalls. Capacity building with good supervision and better remuneration were other suggestions to improve working conditions.

##### 3.2.2.3 Vaccine supply and cold chain

There was clear consensus that there are no vaccine supply shortages in Cox's Bazaar, with the suggestion that this is a priority of the government. Just a few vaccinators described temporary shortages following vaccination campaigns, particularly for the Pentavalent (Penta) and Pneumococcal vaccines (PCV). Further, they explained that sometimes Measles–Rubella (MR) and Tuberculosis (Bacillus Calmette-Guérin - BCG) vaccines are sometimes wasted in fixed-site clinics, due to a lack of children attending.

NGO and government representatives also expressed confidence in the vaccine cold chain. They described well organized and mostly timely delivery from the airport to Cox's Bazar, and storage in government health facilities in line with global standards, emphasizing the use of generators to power the fridges and so manage electricity outages.

“*We have no vaccine supply shortage or interruptions in the supply chain, as this is considered the government's highest priority. The government procures vaccines through UNICEF, placing requests based on demand. Upon arrival at the airport, the vaccines are labeled and received under FDMN then sent directly to Cox's Bazar. The district office distributes the vaccines as per the needs of the camps. Maintaining the cold chain for vaccines is not an issue, and we have ice-lined refrigerators available. We do not experience electricity outages; even if they occur, generators are in place for backup support.”* (Government representative)

A key delivery challenge, raised by NGO representatives, was relying on vaccinators to carry vaccines long distances to outreach posts because transport was not allowed.

##### 3.2.2.4 Vaccination cards

The use of vaccination cards to remind caregivers of vaccination dates and record vaccination was well acknowledged by participants. CHWs emphasized their importance and explained they check the cards during their household visits to remind caregivers about their child's vaccination dates.

“*We visit households the day before vaccination to check the cards and remind mothers to visit the outreach center or fixed-site clinics to vaccinate their children. We have a list of eligible children who are due for vaccination.”* (CHW, Ukhia, high coverage camp)

However, several challenges with the cards were evident. First, there is inconsistency in their use. For example, in Teknaf, CHWs in health facilities give them to caregivers when their child reaches 4 months, while vaccinators in outreach centers hand them out after administering BCG vaccine (immediately after birth). Also, vaccination cards are used as a precondition to receiving ration cards, thereby used to incentivize caregivers to vaccinate their children. CHWs suggested uniform rules are needed for the distribution of vaccination cards to achieve equality. Government representatives proposed this should be at 6 months to avoid early drop out from the vaccination programme.

“*The same vaccine cards some mothers receive within 10 days of vaccinating their children, and some mothers get it after 4 months of vaccination. I suggest having the same rules either to distribute the cards after the BCG vaccine or after receiving four vaccines.”* (CHW, Teknaf, high coverage camp)

Caregivers losing the vaccination cards was viewed as a barrier to them remembering appointments, especially when there are large intervals in the vaccination schedule. Additionally, not bringing the card when visiting the health facility with a child for other purposes meant that vaccinators would not give vaccinations opportunistically without evidence of prior vaccination.

##### 3.2.2.5 Mobilizing caregivers to vaccinate their children

It was evident that a key role of the CHWs and vaccinators is mobilizing caregivers to bring their children for vaccination. These frontline HSPs described how they review children's vaccination records, visit mothers the day before their child's vaccination appointment to remind them to attend, accompany them to the vaccination site on the day of their appointment and telephone them if they do not attend.

Of note was an apparent difference in mobilizing procedures between high and low coverage camps. These differences seemed to be related to the number of CHWs working in a camp but also some CHWs having a designated responsibility for motivating mothers to vaccinate their children, following up with these mothers and taking them to the vaccination site if they missed a scheduled vaccination. This comprehensive approach was assumed to ensure the camp's good vaccination coverage and was suggested as a blueprint for other camps. CHWs from high coverage camps also highlighted that once they heard about the low coverage rates in some camps, they had increased their mobilization efforts which resulted in slight increases in vaccination.

“*Camp variation is high and low because of mobilization. In some camps, they are well-tracked, a cohort has taken a vaccine dosage, the whole cohort was tracked well by the CHW and vaccinators, they follow-up and monitor them regularly and send them for the following scheduled vaccines, but in some camps, follow-up is not good. The mothers who don't go for vaccination were not adequately followed-up*.” (NGO representative)

##### 3.2.2.6 Opportunities for communicating with caregivers about vaccination

It was clear from participants' accounts that communication with caregivers about vaccination happens in many different settings: during household visits by CHWs and vaccinators, in courtyard meetings held by supervisors and Camp in Charge, as part of visits to the health facility/outreach centers and within vaccination campaigns for specific vaccinations. Aside from the campaigns, these vaccination conversations are usually just one part of a wider discussion about different kinds of family health issues, such as post- and antenatal care. Moreover, they are typically only with mothers, rarely with fathers (although this seems to happen more in high coverage camps). CHWs and vaccinators agreed that counseling fathers and household elders is important as they often have an influence on the mother's behavior, but they acknowledged the difficulties in doing this as the men are often not at home when they visit.

“*Husbands do not permit their wives to go for vaccinating for their children. We need to counsel both together, but husbands are not at home, and we cannot counsel them.”* (CHW, Teknaf, high coverage camp)

CHWs emphasized the important role their door-to-door visits and vaccination counseling play in encouraging caregivers to take their children for vaccination and suggested that more could be done. Reflecting on past successes, a CHW from a high coverage camp in Ukhia reported:

“*We worked hard in 2017 when I started to work with this community. I visited the houses every day to counsel the mothers, fathers, and older adults to motivate them. When I visited households door-to-door for the vaccination, they told us to take the vaccine first to see if it harms the body or not. I talked with my supervisor, and then I took the vaccine before them, so they trusted it.”* (CHW, Ukhia, high coverage camp)

Beyond highlighting the importance of one-on-one communication, participants stressed the effectiveness of vaccination campaigns that run at regular intervals every year for different vaccines. In high coverage camps, they were mainly mentioned for Penta and TT (Tetanus Toxoid), and in low coverage camps for measles and diphtheria. CHWs, health facility managers and some government representatives observed that vaccination campaigns in the past had helped to minimize the previously mentioned religious beliefs related to vaccination. Ideas for extending these campaigns included showing videos about the benefits of vaccination at large gatherings (e.g., at big football games or in the local market) and running media campaigns in the caregivers' language. It was noted that extra effort is needed to reach the remote camps.

##### 3.2.2.7 Access to, and treatment at, vaccination sites

Vaccines were reported to be administered to children in fixed-site clinics and outreach points. Scheduled appointments are available on 4–6 days of the week at the fixed-site clinics and on 3 days of the week in outreach posts.

The location of these vaccination sites and difficulties to access them were frequently reported as barriers to vaccination. In fact, key informants hypothesized these were significant reasons for variations in coverage between camps and vaccination sites. CHWs and vaccinators explained how long distances and hilly terrains hinder both caregivers and vaccinators; the latter having to carry heavy loads meaning they often reach outreach posts late. Accordingly, caregivers were described to complain about long waiting times which discouraged them from bringing their children for future vaccinations. This was suggested to confirm some caregivers' existing perceptions of low-quality health care and previous negative experiences when coming to health facilities for other issues.

“*I've observed that some mothers hesitate to bring their children for vaccination due to the distance and location of the outreach post. When the outreach center is situated on a mountain or far from their homes, they are often reluctant to make the journey. Additionally, some fixed-site clinics are quite distant, requiring transportation and incurring costs that some mothers are unwilling to bear. This reluctance poses a challenge in ensuring vaccination coverage for all children in our community.”* (Vaccinator, Teknaf, low coverage camp)

A solution to poor access proposed by NGO representatives was to deliver vaccination in places where refugees regularly go to get food and gas, as well as in nutrition centers where lactating mothers go with their under-2 -year-olds.

#### 3.2.3 Social opportunity

Social opportunity barriers and drivers related to coordination and communication among frontline HSPs and key informants, collaboration with community leaders, relationships between frontline HSPs and the community, and family dynamics.

##### 3.2.3.1 Coordination and communication among frontline HSPs and key informants

There were mixed views among CHWs and vaccinators about their communication and collaboration. CHWs from high coverage camps considered this to be good, citing monthly meetings, regular telephone communication, and sharing information with vaccinators. In contrast, CHWs from low coverage camps were frustrated that vaccinators do not share their lists of eligible children in advance nor advise on how many vaccines were available, meaning that mothers bring children for vaccination when there was insufficient supply.

“*We don't see the vaccinators. I sent 10 mothers for the vaccinations, but seven children received the vaccine, and three couldn't because vaccines were run out. When the mothers return without vaccine, they do not want to go again for the vaccination.”* (CHW, Teknaf, low coverage camp)

Vaccinators were generally more positive, highlighting that they meet regularly with CHWs and liaise closely in following up children who were not attending vaccination. The exception were some vaccinators based at outreach posts who suggested that some CHWs “bad-mouth” outreach posts and encourage mothers to attend the fixed site clinics for vaccination.

Key informants acknowledged that their collaboration is hindered by NGOs competing to demonstrate “good performance,” illustrated by a lack of data sharing, autonomous vaccination programmes and different incentives (e.g., refreshments, sanitation pack) for caregivers. The larger NGOs were criticized for making the branding of initiatives (e.g., the distribution of medical kits) a condition for financial support. Government representatives also disapproved of NGOs following their own protocols for vaccination programmes rather than the Government protocol for the FDMN children.

“*One of the significant challenges in coordination is competition between NGOs. Each organization prioritizes showcasing its performance, leading to reluctance to cooperate with others. This lack of cooperation extends to data sharing, and coordination has deteriorated over time. Issues also persist within government structures, particularly between health officials and administrative officials. Coordination challenges are observed at multiple levels, hindering collaborative efforts.”* (Government representative)

As a positive example of collaboration, both NGO and Government representatives spoke of the well-functioning cold chain which required coordination from multiple players. A Government representative suggested that WHO and UNICEF should take the lead on vaccination due to their experience in the Expanded Programme on Immunization (EPI). NGO participants commented that good collaboration relied on them having good relationships with the Civil Surgeon and clear roles and responsibilities for all partners.

##### 3.2.3.2 Collaboration with community leaders

The important role of community leaders, particularly majees (block leaders), imams (religious leaders) and Camp-in-Charge in promoting childhood vaccination was well recognized by participants. They explained how majees do miking to remind caregivers to take children for vaccination, accompany vaccinators on households visits and hold outreach posts at their houses. The main role of imams was seen to be miking from the mosque. Camp-in-Charges were mentioned to promote childhood vaccination in their meetings with community members. All were perceived to be trusted by the community and influential with potential for them being more involved in promoting vaccination:

“*We do miking through the Maji as they trust them. When the Maji announce over the miking, they trust them more.”* (Vaccinator, Teknaf, low coverage camp)

Ideas included majees running awareness raising sessions as part of their regular community meetings and imams encouraging vaccination during Friday prayers. Just a few frontline HSPs had reservations. Some CHWs voiced concerns that majees sometimes pressurize caregivers to bring children for vaccination rather than encouraging them. Some vaccinators had found imams to be uncooperative citing their own misconceptions about vaccination.

##### 3.2.3.3 Relationships between frontline HSPs and the community

There was clear agreement amongst CHWs that they have a good relationship with the community.

“*Our relationship with the community is friendly and built on trust, as we visit them every day. Over time, we become like family members to them, and this close bond fosters trust between us and the mothers, which is essential for our work.”* (CHW, Ukhia, low coverage camp)

Conversely, vaccinators reported that they may experience misbehavior from caregivers during door-to-door visits, with some even fearing violence.

“*I've encountered situations where mothers exhibit misbehavior during home visits to counsel them for vaccination. This often stems from their frustration with waiting long periods at health facilities to receive vaccines. Some mothers even express extreme reluctance, stating that they would refuse vaccination even if it meant risking their lives.”* (Vaccinator, Teknaf, low coverage camp)

Gender seemed to play a role concerning the relationship between frontline HSPs and community members. One male CHW and some male vaccinators described how they face difficulties during household visits with young mothers, who are reluctant to speak with a man due to “Purdah” (the seclusion of women from public observation). CHWs no longer found this to be a problem once they are known to the families.

##### 3.2.3.4 Family dynamics

Family dynamics was believed to be an important barrier to vaccination. Participants described how mothers would miss vaccination appointments because they prioritized household work or were concerned to leave other children at home unattended. Gender-related power interactions were also observed, particularly by the frontline HSPs, with some mothers needing permission of the child's father to take the child for vaccination, some fathers refusing vaccination if their child had previously experienced side effects, and mothers even facing violence from the child's father for both these events.

”*We face the challenge to motivate mothers for vaccination after the third dose of the Pentavalent vaccine. The children suffer from fever and pain after this vaccination. Sometimes mothers are beaten by their husbands when their child gets sick due to the vaccine. So they do not want to go for the next scheduled vaccine due to these reasons.“* (CHW, Teknaf, low coverage camp)

#### 3.2.4 Motivation

The motivation barriers and drivers related to HSPs' confidence in the benefits of vaccination and caregivers' concerns and lack of trust.

##### 3.2.4.1 Positive attitudes of HSPs toward vaccination

HSPs expressed themselves to be convinced of the benefits and importance of vaccination without exception. Frontline HSPs showed to be confident about the different benefits of vaccination and the risks of not vaccinating (as described under Capability), emphasizing the prevention of VPDs and highlighting the safety of vaccines with only minor side-effects. Key informants spoke about ”uncountable benefits of vaccination“ including the eradication of VPDs, and the protection of the host community.

##### 3.2.4.2 HSPs' views on vaccine eligibility

Overall, participants supported the existing age limit for vaccine eligibility of 2 years. They recounted difficulties in mobilizing older children for vaccination outweighing administering vaccines to them. The only group that expressed a different opinion regarding the age of eligibility were the NGO informants who suggested increasing the eligibility age to 5 years as they thought it could improve coverage.

“*I recommend that the eligibility age for vaccinations could be flexible, extending up to 5 years, specifically, I suggest expanding the age range for diphtheria and measles vaccines, mainly due to prevalent cases within the camps. Increasing the age range can include more children in vaccination efforts.”* (NGO representative)

##### 3.2.4.3 Caregivers' concerns and lack of trust

Participants spoke of motivational barriers they observe in caregivers relating to concerns about side effects, Purdah, and lack of trust in the health system. All were perceived to be more prevalent in low coverage camps and among fathers, grandparents, and the less educated community members. Concerns focused on minor side effects including fever, pain, swelling, and sleep disturbance, and seemed to be particularly prevalent for the third dose of the Pentavalent vaccine which is a combined 5-in-1 vaccination. Also, some mothers or older daughters were reported to be reluctant to leave the house to take a child to get vaccinated due to concerns to maintain Purdah. It was suggested that being previously excluded from vaccination programmes in Myanmar increased some caregivers' skepticism toward vaccination. This previous negative experience with health services was then reconfirmed when medicines (including vaccines) were unavailable.

“*It is challenging to convince fathers and more religious members to allow their wives or daughters-in-law to leave home for vaccination. Some fathers or fathers-in-law do not allow their wives or daughters-in-law to leave home for the vaccine due to household chores, and the child may get sick after vaccination. Additionally, wives face violence from husbands if the child falls sick after vaccination without their permission. Older adults are more resistant, citing their own good health without vaccination when they were in Myanmar.”* (CHW, Teknaf, low coverage camp)

## 4 Discussion

This paper presents a qualitative study on the barriers and drivers to childhood vaccination in Cox's Bazar, one of the largest refugee camps worldwide. To our knowledge, this is the only qualitative study to focus solely on HSPs' perspectives on childhood vaccination in Cox's Bazar. The study applied a purposive approach to sampling which allowed us to capture different perspectives and compare camps with high and low vaccination coverage. Further, it used the COM-B framework to follow a theory-informed approach. Allover, this paper presents the perspectives of HSPs on their “delivery side” of vaccination, and the role of caregivers in this process.

The findings showed that multiple interlinked individual and context factors influence the delivery of childhood vaccination in Cox's Bazar. This closely aligns with the findings of a scoping review conducted in Cox's Bazar (16) and parallels the multifaceted influences on effective immunization delivery in refugee camps, reported more widely (31). Of note was the high level of agreement amongst the different professional groups on the key barriers and drivers. This detailed understanding of different types of HSPs' perspectives on delivering vaccination seems under-explored in the wider refugee camp vaccination literature. The insights presented in this paper provide valuable direction for designing tailored interventions to improve vaccination coverage in Cox's Bazar and may also have relevance for vaccination initiatives in other refugee camps.

On an individual level among HSPs, knowledge gaps acted as barriers, while detailed knowledge of the purpose of vaccination, good communication skills and confidence toward the benefits of vaccination served as drivers. For caregivers, a lack of awareness of vaccination and concerns about side effects alongside mistrust were, from HSP's perspectives, the main issues hindering take-up of vaccination. Contextual barriers included information system deficiencies, challenging family dynamics, poor working conditions of HSPs, and inaccessibility of vaccination sites; while effective communication between all parties, mobilization strategies and incentives acted as drivers.

Very few, but potentially important, differences in the barriers and drivers of delivering vaccination were identified between high and low coverage camps. Communication and collaboration between the different HSP professional groups and organizations working with vaccination in the camps, HSPs' vaccination knowledge, and the way and intensity in which CHWs track, remind and mobilize caregivers were all seen to be better in high coverage camps. Caregivers' vaccination concerns and mistrust, as well as difficulties in accessibility were found to be more prevalent in low coverage camps. In the following sections, we focus on these differences and offer solutions, which, if effectively tackled, seem to have considerable potential to improve childhood vaccination coverage in Cox's Bazar.

### 4.1 Effective collaboration between different groups of HSPs

Several challenges appeared to hinder effective collaboration between the different stakeholders involved in vaccination in the camps. Serious communication gaps between the government and different NGOs, as well as between CHWs and vaccinators were repeatedly mentioned. Also, rather than coordinating efforts via meetings that involve all stakeholders, each of the different organizations seemed to liaise with the government individually. It was suggested that every group wants to prove their superiority, thereby increasing their legitimacy, instead of effectively cooperating for the same goal. This is not a new challenge in Cox's Bazar nor unique to immunization services. Indeed, the UNHCR reported how “competing centers of authority leading to service fragmentation” (32) hindered the emergency response at the start of the influx of Rohingya refugees into Bangladesh. There also seemed to be a reluctance to share data, as organizations were uncertain about the accuracy of their coverage metrics. The literature on public health surveillance shows that sharing surveillance data improves disease detection and response (33, 34). This means that monitoring (low) vaccination coverage rates in certain vaccines or areas can help to prevent outbreaks. Given the numerous NGOs operating in Cox's Bazar, one key challenge thus lies in harmonizing vaccine surveillance and vaccination programs effectively. Effective collaboration amongst CHWs and vaccinators, different NGOs and the government is vital for efficiently using existing efforts and resources. Especially when working in emergency and low resource settings, overcoming competition and other barriers to collaboration and engaging stakeholders in discussions with each other is crucial for a unified and efficient approach (35). To this end, a consistent recommendation from key stakeholders such as the WHO (31), UNHCR (32), UNICEF (21) is to clearly specify each organization's role and accountability with a stated leadership structure.

### 4.2 Tracking, reminding, and mobilizing caregivers

Our study revealed that effective strategies for mobilizing caregivers to vaccinate their children include regular vaccination campaigns and the proactive role of frontline HSPs in their interaction with caregivers, e.g., through household visits. The importance of trusted health workers and the effectiveness of reminders on caregivers' vaccination behaviors are both well reported for migrant (36, 37) and non-migrant populations (19, 38). Tracking vaccination and reminding caregivers of vaccinating their children is crucial when aiming to achieve high vaccination coverage (31). The difficulties of doing so have been reported elsewhere with regard to vaccinations that are scheduled with larger intervals. For example, the drop-out in vaccination against measles (mumps), rubella (MR or MMR) shows to be a worldwide trend, and is particularly pronounced in resource poor settings (39). Also, the effectiveness of follow up for second and third dose vaccinations that are administered in large intervals has been shown in previous vaccination campaigns in Cox's Bazar (9). The findings presented in this paper corroborate this evidence as they show that in order to ensure full vaccination coverage, it is important that caregivers are followed up, ideally in person by trusted frontline HSPs. The study, however, also revealed how tracking (e.g., children eligible for vaccination) can be difficult due to inadequate record-keeping, emphasizing the need for improved structures and collaboration in the camps in Cox's Bazar.

The distribution of vaccination cards linked to incentives such as hygiene kits or medication (Panadol) to caregivers after their child's vaccination have been used as a strategy to increase childhood vaccination coverage without strong evidence of their effectiveness (40, 41). Our findings suggest they may be an effective measure, but importantly, their use should be consistent throughout camps in order to achieve equality. Moreover, the use of vaccination cards and incentives in refugee and low resource settings, particularly when combined with food rations or other products that are essential to daily life, needs to be considered carefully from an ethical point of view (42).

### 4.3 The importance of frontline HSPs' knowledge and communication skills

Considering the important role that HSPs play in tracking, reminding and mobilizing caregivers, it is not surprising that their knowledge and skills were seen to be a key to achieving good vaccination coverage amongst all groups of participants. Crucially, HSPs from high coverage camps demonstrated a more comprehensive and precise understanding of vaccination coverage, VPDs and the benefits of vaccination. Thus, it may be assumed that they are able to communicate even more confidently and accurately to caregivers and other relevant community members. This underscores the importance of in-depth knowledge and communication skills of those delivering childhood vaccination, a factor which is not unique to our study, but reflected in the literature on childhood vaccination generally (19, 43) and in other refugee settings (31, 41). This finding suggests that refining or supplementing the training which frontline HSPs receive in Cox's Bazar might help to improve childhood vaccination coverage. Apart from a need of well-trained HSPs in all camps, findings showed there is a pressing need for a more equitable distribution of the workforce. CHWs in particular were described to carry a high burden of work not only related to vaccination but also to many other health-related issues, which again is enhanced in bigger camps with long distances and difficulties in accessibility.

Further, our research reveals that specific community members, including fathers, older adults, those with lower education levels, and some community leaders, are perceived to be more hesitant toward vaccination. Addressing this challenge requires boosting HSPs' knowledge related to the specific concerns raised by these groups and training HSPs in effectively targeting and communicating with these individuals, addressing their concerns and overcoming reluctance. This aligns with established principles in the health communication literature which affirm that tailoring communication strategies to the addressee is of crucial importance (44). It also confirms participants' reports and a previous qualitative study (45) that highlighted the success of earlier Cox's Bazar vaccination campaigns in addressing religious beliefs leading to a positive shift in perceptions and helping counter the negative impact of such beliefs on vaccination.

### 4.4 Mistrust toward vaccination in social and historical context of FDMN/Rohingya refugees

A frequently mentioned barrier to vaccination was caregivers' lack of trust. Related to this, the Rohingya's long history of exclusion and persecution in Myanmar needs to be considered, which was primarily driven by the government's discriminatory policies in Myanmar that extended to their access to healthcare, education, and citizenship rights (46). This exclusion might have contributed to a profound mistrust among the Rohingya toward not only the healthcare system but all forms of government structures. Trust forms a key element in enhancing health in a society (or community) (47). This finding aligns with current literature, i.e., a recent review on under-immunization in refugee and migrant populations (37), and a study on health beliefs and barriers to healthcare of Rohingya refugees based in the US (48). While the participants in this study reported how vaccination campaigns and effective communication in Cox's Bazar in the past years had helped to alleviate religious concerns around vaccination, remaining vaccine hesitancy (or refusal) might partly be due to caregivers' lifelong beliefs and norms and a sense of mistrust, and might therefore not be easy to change. Future vaccination delivery in Cox's Bazar should continue to proactively address sociocultural issues and beliefs in order to enhance the effectiveness of vaccination campaigns (45).

### 4.5 Limitations

This study has a number of limitations. First, the sample of participants might mean that the views of certain stakeholders and HSPs were not considered. However, we sampled with a view to obtaining a diversity of views and practices, and data saturation was achieved across the COM factors. Also, the comparative analysis and triangulation across participant groups gives us confidence in having captured the main points. Limitations further include that in some FGDs, a WHO representative was present, which may have led to some socially desirable responses. Further, the data that was collected did not allow a differential analysis of HSPs' views on different vaccines, but only insights on barriers and drivers to the delivery of the childhood vaccination overall. We did a differentiation where possible (e.g., for MMR 1 and 2) but apart from this, findings seemed to apply for all vaccinations. Also, we only investigated HSPs, not caregivers themselves. Accounts of caregivers' concerns and views are therefore only provided from the HSPs' perspectives. Our findings could be triangulated with information on different childhood vaccines and the position of FDMN/Rohingya caregivers to gain an even more comprehensive understanding. In fact, we are in the process of triangulating the qualitative findings presented in this paper with survey findings collected from around 900 caregivers in the FDMN/Rohingya refugee camps. This will inform the development and design of tailored and targeted interventions for better childhood vaccination coverage in the camps of Cox's Bazar.

## 5 Conclusions

HSPs experience many inter-related barriers and drivers to the delivery of the childhood vaccination in Cox's Bazar. For increasing childhood vaccination in the camps that currently have low vaccination coverage, context-related changes seem necessary, like improved and effective collaboration between the different organizations and stakeholders that are involved in childhood vaccination, a well-trained and equally distributed health workforce especially in areas that are difficult to access, and an ethically responsible use of incentives and vaccination cards. On an individual level, targeted communication and campaigning might be further useful in lowering vaccine hesitancy, particularly if mistrust and socio-cultural barriers are addressed. By combining interventions that tackle these individual and context factors, childhood vaccination in the world's largest refugee camp might be improved and VPD-related morbidity and mortality reduced.

## Data availability statement

The raw data supporting the conclusions of this article will be made available by the authors, on reasonable request to the principal investigator.

## Ethics statement

The study received ethical approval from the Institutional Review Board (IRB) of the Cox's Bazar Ethics Committee, the Research Review Committee Bangladesh at WHO Bangladesh as well as WHO South-East Asia (SEARO). The study was conducted in accordance with local legislation and institutional requirements. Written informed consent for participation in this study was provided by the participants.

## Author contributions

SR: Conceptualization, Formal analysis, Methodology, Project administration, Writing – original draft, Writing – review & editing. HW: Conceptualization, Formal analysis, Methodology, Writing – original draft, Writing – review & editing. SA: Data curation, Formal analysis, Writing – review & editing. BK: Conceptualization, Funding acquisition, Supervision, Writing – review & editing. JM: Supervision, Writing – review & editing. AS: Data curation, Funding acquisition, Investigation, Project administration, Writing – review & editing. CJ: Conceptualization, Formal analysis, Funding acquisition, Methodology, Supervision, Writing – original draft, Writing – review & editing.
