# Supplementary material for: Health service providers' views on barriers and drivers to childhood vaccination of FDMN/Rohingya refugees: a qualitative study in Cox's Bazar, Bangladesh
Source: Front Public Health. 2024 Jul 9;12:1359082. doi: 10.3389/fpubh.2024.1359082 (PMC11265221; doi:10.3389/fpubh.2024.1359082)
Supplement: Supplementary file 1 [file Data_Sheet_1.docx]

Supplementary Material

Health service providers’ views on barriers and drivers to childhood vaccination of FDMN/Rohingya refugees: A qualitative study in Cox's Bazar, Bangladesh

Sarah Reda^1*^, Heide Weishaar^1*^, Sadika Akther^2^, Basel Karo^1^, Jorge Martínez^3^, Aarti Singh^3^, Cath Jackson^4^

^1^ Centre for International Health Protection, Robert Koch Institute, Nordufer 20, 13353 Berlin, Germany

^2^ School of Health & Social Development, Deakin University, Burwood, Victoria, Australia

^3^ World Health Organization Emergency Sub-Office in Cox’s Bazar, Bangladesh

^4^ Valid Research Ltd, Sandown House, Sandbeck Way, Wetherby, West Yorkshire, LS22 7DN, UK

*These authors share first authorship

**Supplementary Data**

# Discussion guide for FGDs with vaccinators and for interviews with health facility managers

INTRODUCTION:

*Moderator:* Hello and thank you for talking with us today. My name is ... and this is (name) who is assisting me to translate, record and take notes of your responses. We work at Cox’s Bazar Medical college and /or WHO Office Cox’s Bazar

We are asking for help from health service providers like you who are involved in childhood vaccination to understand barriers and drivers to delivering childhood vaccination in Cox’s Bazar. We are particularly interested in hearing your experience and views.

We will be audio-recording recording and taking notes on the conversation for research purposes only. We will not use any personal information that can identify you in our analysis or report.

There are no wrong answers. You are the experts, and we will learn from you.

**Instructions for FGDs only (not for the interviews with health facility managers)**

Everybody’s input is important, and we hope everybody will join in the discussion. We hope to create an atmosphere of acceptance of each other’s views and practices which might or might not be different. We have a few guidelines for us to follow to make our discussion inclusive and productive for everyone.

1. Please switch off your mobile phones or put them on silent.
2. Please do not talk at the same time as someone else or interrupt so that we can hear each of you without difficulty.
3. If you want to say something, please wait until the person speaking is finished. If someone else begins to talk before you, do not worry. I (the moderator) will ask each time if anyone else wants to comment before moving on to the next question.
4. Please do not start separate discussions among yourselves. We want everyone to be a part of one discussion only.
5. This is an open discussion, and we expect you to be respectful but please don’t judge, everyone’s opinion is valid.
6. Everything said in this discussion should remain confidential and should not be shared outside the group.

Do you have any questions? Let’s begin.

| **INTRODUCTORY (WARM UP) QUESTION** |
| --- |
| **Can you tell me briefly about your role in childhood vaccination in Cox’s Bazar?**  *Prompts*   - What do you do? - Which camp do you work in? |

| **CHILDHOOD VACCINATION COVERAGE AND VACCINATION PREVENTABLE DISEASES IN COX’S BAZAR** |
| --- |
| **Do you know the childhood vaccination coverage data for the camp you work in?**  *Prompts*   - How does this compare to other camps in Cox’s Bazar? - Why do you think the coverage in your camp is higher/lower than for other camps?   **Do you know how many children in your camp are not fully vaccinated by 2 years of age (and so are no longer eligible for vaccination?)**  *Prompts*   - How does this compare to other camps in Cox’s Bazar? - Why do you think this is higher/lower than for other camps?   **Do you know the Vaccine Preventable Diseases data for the camp you work in?**  *Prompts*   - Are you notified of these cases (e.g., measles, diphtheria) in your camp? How? |

**I’d like to talk today about some things that might influence childhood vaccination practices.**

| **YOUR THOUGHTS ON CHILDHOOD VACCINATION** |
| --- |
| **Let’s start with your own thoughts on vaccines and vaccination.**  **What are your thoughts on childhood vaccination?**  *Prompts*   - What do you think are the benefits of vaccinating children in Cox’s Bazar? - What do you think are the risks of vaccinating children in Cox’s Bazar? - What do you think of the system that once children are 2 years of age, they are no longer eligible for the vaccinations they have missed?   **What or who do you think influences your opinion about vaccination?**  *Prompt*   - How do these things (people, norms, specific information, etc.) influence your opinion? |

| **COMMUNICATION ABOUT CHILDHOOD VACCINATION** |
| --- |
| **I would now like to know what is communicated about childhood vaccination in the camp you work in.**  **How, if at all, is childhood vaccination promoted to caregivers?**  (*Caregiver is any person taking care of children and directly responsible for their health needs e.g., mother, father, aunt, uncle, grandfather, grandmother or anyone in the household who is 18 years of age or an emanicipated minor*.)  *Prompts*   - Are any childhood vaccination campaigns run? Please tell me about them. - Do caregivers learn about childhood vaccination when they visit a health facility? Or outreach post? How does this happen? (e.g. information in the waiting room, conversations with frontline workers)   **Do you personally talk to people you see in your day-to-day work about childhood vaccination?**  *Prompts*   - Do you talk to anyone routinely or is it spontaneous whether you talk to someone who uses the services about childhood vaccination or not? - Who do you talk to? Why do you talk to these people particularly? - Do you talk to other people than caregivers about childhood vaccination? By caregivers I mean people who take care of a child under the age of 2.   **When you talk to people about childhood vaccination, what do you talk about?**  *Prompts*   - Do you provide written information on childhood vaccination? If so, what kind of information? - Do you recommend childhood vaccination? Why/Why not? - Do people tell you about why they don’t think children should be vaccinated? How do you respond to these concerns? - How confident are you in talking to caregivers who are hesitant to get their children vaccinated? - How confident are you in talking to caregivers who decline vaccination for their children?   **How do you think childhood vaccination could be better promoted in your camp?**  *Prompts*   - Who else do you think could promote childhood vaccination with caregivers? - Should specific groups be targeted more? Which ones? - Do you think there are specific caregiver concerns that should be addressed when promoting childhood vaccination? - What can be done to reassure caregivers to vaccinate children? - What would help you for your conversations with caregivers specifically about childhood vaccination? |

| **VACCINATION PROCESSES** |
| --- |
| **Next, I want to better understand the process of how children receive vaccination in the camp you work in?**  **How do the vaccination procedures work?**  *Prompts*  Where do children get vaccinated?   - How is this location decided?   What is the process of getting a child vaccinated in the health facility/outreach post that you work in?   - Are caregivers invited to bring their child for vaccination? If so, how and when? - When can children receive vaccination? Is it every day, once a week, less often? - Are appointments scheduled? - Do you do vaccination spontaneously when when a caregiver comes with the child for another reason, e.g., nutrition, treatment of other ailments, accompanying an adult? Why/why not? - Do you have sufficient time in a working day to do vaccinations? Why/why not?   **In your experience, why do some children not receive all recommended vaccinations?**  *Prompts*   - Do you always have sufficient vaccines? If not, how often do shortages occur? What do you do when there is shortage? - Are children less likely to receive certain types of vaccinations? If so, which ones? Why those? - What do caregivers say about single and multiple/combination vaccinations - Children can only receive vaccination if they are under the age of 2. Does this cause any problems or not? - Are there certain types of children who are less likely to receive the recommended vaccinations? - Are there certain types of caregivers who are likely to not vaccinate their children?   **Imagine a caregiver wants to bring their child to be vaccinated. What could be challenges they face in doing so?**  *Prompts*   - Might they not know when to come? - Might they not know where to come? - Might they have access challenges? - Might they have competing commitments? |

| **IDEAS FOR IMPROVING COVERAGE** |
| --- |
| **In your opinion, what could be done to improve vaccination procedures in the camp you work in?**  *Prompts*   - Can vaccination procedures be improved in the camp? How? - How could the challenges be overcome that you have talked about? - What is the most important action that needs to happen to increase uptake of childhood vaccines in the camp?   **What do you think is done well in the camp you work in that could also be done in other camps to improve childhood vaccination?** |
|  |
| **FINAL QUESTION** |
| **Is there anything else about childhood vaccination that you think is important to say before we finish?** |

# Discussion guide for FGDs with community health workers

**INTRODUCTION:**

*Moderator:* Hello and thank you for talking with us today. My name is ... and this is (name) who is assisting me to translate, record and take notes of your responses. We work at Cox’s Bazar Medical college and /or WHO Office Cox’s Bazar.

We are asking for help from health service providers like you who are involved in childhood vaccination to understand barriers and drivers to delivering childhood vaccination in Cox’s Bazar. We are particularly interested in hearing your experience and views.

We will be audio-recording and taking notes on the conversation for research purposes only. We will not use any personal information that can identify you in our analysis or report.

There are no wrong answers. You are the experts, and we will learn from you.

Everybody’s input is important, and we hope everybody will join in the discussion. We hope to create an atmosphere of acceptance of each other’s views and practices which might or might not be different. We have a few guidelines for us to follow to make our discussion inclusive and productive for everyone.

1. Please switch off your mobile phones or put them on silent.
2. Please do not talk at the same time as someone else or interrupt so that we can hear each of you without difficulty.
3. If you want to say something, please wait until the person speaking is finished. If someone else begins to talk before you, do not worry. I (the moderator) will ask each time if anyone else wants to comment before moving on to the next question.
4. Please do not start separate discussions among yourselves. We want everyone to be a part of one discussion only.
5. This is an open discussion, and we expect you to be respectful but please don’t judge, everyone’s opinion is valid.
6. Everything said in this discussion should remain confidential and should not be shared outside the group.

Do you have any questions? Let’s begin.

| **INTRODUCTORY (WARM UP) QUESTION** |
| --- |
| **Can you tell me briefly about your role in child vaccination in Cox’s Bazar?**  *Prompt*   - What do you do? - Which camp do you work in? |

| **YOUR THOUGHTS ON CHILDHOOD VACCINATION** |
| --- |
| **What do you think about childhood vaccination?**  *Prompts*   - What do you think are the benefits of vaccinating children in Cox’s Bazar? - What do you think are the risks of vaccinating children in Cox’s Bazar?   **What or who influences your opinion about childhood vaccination?**  *Prompt*   - How do they influence you? |

| **PROMOTING CHILDHOOD VACCINATION** |
| --- |
| **How, if at all, is childhood vaccination promoted to caregivers in your camp?**  (*Caregiver is any person taking care of children and directly responsible for their health needs e.g., mother, father, aunt, uncle, grandfather, grandmother or anyone in the household who is 18 years of age or an emanicipated minor*.)  *Prompts*   - Are any childhood vaccination campaigns run? Please tell me about these. What is your role in these campaigns? - Do caregivers receive information about childhood vaccination when they visit a health facility? Or an outreach post? Please tell me about this.   **How do you think childhood vaccination could be better promoted in your camp?**  *Prompts*   - Who else do you think could promote childhood vaccination to caregivers? - Should specific groups of children be targeted more? Which ones? |

| **VISITING HOUSEHOLDS** |
| --- |
| **I’d like to better understand what happens when you visit households.**  **Approximately how many households do you visit in a week? *(ask per day if easier)***  **Approximately how many times in a week do you discuss child vaccination with these households?** *(ask per day if easier)*  *Prompts*   - Who do you talk to about childhood vaccination during these household visits? - What do you talk about?   **Do you meet caregivers who have concerns about having their child vaccinated?**  *Prompts*   - What concerns do they have? - How do you respond to these concerns? - How confident are you in having these conversations?   **Do you meet caregivers who refuse to have their child vaccinated?**  *Prompts*   - What are their reasons for refusing? - How do you respond to this? - How confident are you in having these conversations?   **What would help you with your conversations with caregivers about childhood vaccination?**  **What can be done to reassure caregivers to vaccinate their children?** |

| **MOBILISING FAMILIES TO ATTEND FOR VACCINATION** |
| --- |
| **I’d like to know more about what happens when you advise a caregiver that their child is due to be vaccinated.**  **How do you know when a child is due to be vaccinated?**  *Prompts*   - Do you have access to children’s vaccination records that are kept in the health facility/outreach post? If not, what information do you have that can tell you about a child due to be vaccinated? - Do you have the microplans?   **Where do you advise caregivers to go for childhood vaccination?**  *Prompts*   - Do you advise them to go to a health facility? to an outreach post? Or somewhere else? (*Ask about each of these separately*) - How do you decide on whicho f these places to send them to? - How do you know where the health facility? Where the outreach posts are? or the other places you mentioned? (*Ask about each of these separately*)   **Do you know the vaccinators from the health facilities? and from the outreach posts?** (*Ask about each of these separately*)  **In your opinion, how good is the co-ordination between you as CHWs and the vaccinators in the health facilities? In the outreach posts?** (*Ask about each of these separately*) Why do you think that? What are the challenges?  *Prompt*   - Do you share information about childhood vaccination with each other? Why/why not?   **Imagine a caregiver wants to take their child to be vaccinated. What could be challenges they face in doing so?**  *Prompts*   - Might they not know when to go? - Might they not know where to go? - Might they have some access challenges? - Might they have competing commitments or priorities? |

| **IDEAS FOR IMPROVING COVERAGE** |
| --- |
| **In your opinion, what could be done to improve vaccination coverage in the camp you work in?**  *Prompts*   - How could the challenges that you have talked about be overcome? - In your opinion, what is the most important action that needs to happen to increase uptake of childhood vaccines in the camp?   **What do you think is done well in the camp you work in that could also be done in other camps to improve childhood vaccination?** |

| **FINAL QUESTION** |
| --- |
| **Is there anything else about childhood vaccination that you think is important to say before we finish?** |

# Discussion guide for interviews with key informants for childhood vaccination (excluding health facility managers)

**INTRODUCTION:**

*Moderator:* Hello and thank you for talking with us today. My name is ... and this is (name) who is assisting me. We work at Cox’s Bazar Medical College and /or WHO

We are asking for help from people like you who have strategic, management or administrative responsibility for the childhood vaccination programme in Cox’s Bazar or who works as a key partner in the childhood vaccination programme. We are particularly interested in hearing your experience and views so we can understand barriers and drivers to delivering childhood vaccination in Cox’s Bazar. There are no wrong answers. You are the expert, and we will learn from you.

We will be audio-recording and taking notes on the conversation for research purposes only. We will not use any personal information that can identify you in our analysis or report. If you would prefer not to be recorded, please say and we can just take notes.

Do you have any questions? Let’s begin.

| **INTRODUCTORY (WARM UP) QUESTION** |
| --- |
| **Can you tell me briefly about your role in child vaccination in Cox’s Bazar?**  *Prompt*   - What do you do? - Which camp(s) do you work with? |

| **CHILDHOOD VACCINATION COVERAGE AND VACCINATION PREVENTABLE DISEASES IN COX’S BAZAR** | |
| --- | --- |
| **Do you know the childhood vaccination coverage data for Cox’s Bazar? (Ukhiya, Teknaf)**  *Prompts*   - What is your assessment of this coverage? (Good, poor etc) - Does it vary by camp? Why do some camps have high coverage and others have low coverage?   **Do you know how many children in Cox’s Bazar (Ukhiya, Teknaf) are not fully vaccinated by 2 years of age (and so are no longer eligible for vaccination?)**  *Prompts*   - What is your assessment of these data? (Good, poor etc) - Does it vary by camp? Why?   **Do you know the vaccine preventable diseases data for Cox’s Bazar? (Ukhiya, Teknaf)**  *Prompt*   - Are you notified of these data (e.g., measles, diphtheria cases)? How? |  |

**I’d like to talk today about some factors that might influence childhood vaccination practices.**

| **YOUR THOUGHTS ON CHILDHOOD DISEASES AND VACCINATION** |
| --- |
| **Which vaccine preventable diseases do children come to the health facilities with?**  *Prompt*   - Any disease that you are particularly concerned about?   **What are your thoughts on childhood vaccination?**  *Prompts*   - What do you think are the benefits of vaccinating children in Cox’s Bazar? - What do you think are the risks of vaccinating children in Cox’s Bazar? - What do you think of the system that once children are 2 years of age, they are no longer eligible for the vaccinations they have missed?   **What or who do you think influences your opinion about vaccination?**  *Prompt*   - Why do these things (people, norms, specific information, etc.) influence your opinion? |

I’d like to talk next about vaccination processes.

| **VACCINATION PROCESSES** |
| --- |
| **How is childhood vaccination organised in Cox’s Bazar? (Ukhiya, Teknaf)**  *Please can you describe these processes to me.*   - Ordering vaccines - Delivery of vaccines to the camps - Storage of vaccines - What types of health facilities administer the vaccinations - Which staff administer the vaccinations - How identify children who are due for vaccination - How invite the caregivers to bring their child - How motivate caregivers to bring their child (e.g. campaigns, information) - How mobilise caregivers to the vaccination sites - How record/track vaccination status of children - How link vaccination with other services   **What are the main challenges associated with any of these procedures?**  **What are your ideas for solutions?** |
| **Which organisations are involved in the procedures we just discussed?**  *Prompts*   - How well do the organisations work together? - What are the challenges?   **What are your ideas for improving the collaborative working across organisations?** |

| **In your experience, why do some children not receive all recommended vaccinations?**  *Prompts*   - Are there some caregivers who do not know when to bring their children? Or where to come? - Are there always have sufficient vaccines? If not, how often do shortages occur? What happens when there is shortage? - Are children less likely to receive certain types of vaccinations? If so, why? - Children can only receive vaccination if they are under the age of 2. Does this cause any problems or not? - Are there certain types of children who are less likely to receive the recommended vaccinations? - Are there certain types of caregivers who are likely to not vaccinate their children? - Are there certain types of camps where this happens more?   **Imagine a caregiver wants to bring their child to be vaccinated. What could be challenges they face in doing so?**  *Prompts*   - Might they not know when to come? - Might they not know where to come? - Might they have access challenges? - Might they have competing commitments? |
| --- |

| **IDEAS FOR IMPROVING COVERAGE** |
| --- |
| **What is the most important action that needs to happen to increase uptake of childhood vaccination in Cox’s Bazar? (Ukhiya, Teknaf)**  **What do you think is done well in some camps or health facilities that could also be done in other camps or facilities to improve childhood vaccination?** |
| **FINAL QUESTION** |
| **Is there anything else about childhood vaccination that you think is important to say before we finish?** |

# Example RAP sheet

| Example RAP sheet - Caregiver challenges | | | | |
| --- | --- | --- | --- | --- |
|  | High coverage (camp 1W) | | Low coverage (camp 19) | |
|  | Summary of discussion | Interesting discussion and good quotes (note minutes in audio) | Summary of discussion | Interesting discussion and good quotes (note minutes in audio) |
| Practical challenges for caregivers? |  |  |  |  |
| Know WHEN to come? | The  CHWs said the mothers do not know the exact date. They go to the mothers before the day of the scheduled vaccine to remind them about the date to go for the vaccination. |  | If the CHWs said the mothres do not know and they go to the mothers before the day of the scheduled vaccine to remind the mothers when to go for the vaccination. |  |
| Know WHERE to come? | CHWs said that mothers know where to go for the vaccine, still  the CHWs go to the mothers before the day of the scheduled vaccine to remind them about the place to go. |  | CHWs said that mothers sometime forget where to go for the vaccine if the CHWs do not go to the mothers before the day of the scheduled vaccine to remind them about the place to go. |  |
| Access challenges? | The CHWS mentioned the distance between the outreach centre or fixed site and some blocks on the hill. The mothers like to go to the fixed-site clinics or outreach posts close to their blocks. The mothers do not want to go for the vaccination because of the distance and mountain. |  | The CHWS mentioned that mothers do not come to the outreach centre or fixed site due to the distance. Some blocks are situated on the hill, so mothers do not want to climb the hills for vaccination. The mothers who live near the fixed-site clinics come for the vaccine, but those who live far, do not want to come for the vaccine. |  |
| Family commitments? | The CHWS mentioned the distance between the outreach centre or fixed site and some blocks on the hill. The mothers like to go to the fixed-site clinics or outreach posts close to their blocks. The mothers do not want to go for the vaccination because of the distance and mountain. One CHW said the mothers want the vaccination if the vaccinators are female, especially the wives of the religious leaders who maintain Parda. |  | All CHWs said that the mothers always refuse to go for the vaccine because of their household work, taking care of children who get sick after the vaccination. |  |
| Family member permission? | When the CHWs go to the mothers to send them to the outreach post for the vaccination, the mothers call their husbands for permission to go for the vaccination. |  | All CHWs said that mothers sometimes do not go for the vaccination for their children because their husbands do not permit them to go. |  |
| Other practical barriers? | No comment on this |  | No comment on this |  |
| Other |  |  |  |  |

# Data Availability Statement

The full datasets (RAP sheets of FGDs and interviews) are available on request.
